# A retrospective descriptive study of male perpetrators of intimate partner violence referred by judicial authorities: an example from Turkey

**DOI:** 10.1007/s00737-024-01495-5

**Published:** 2024-07-18

**Authors:** Şeyma Sehlikoğlu, Ahmet Nalbant, Kerem Sehlikoğlu, Behice Han Almiş

**Affiliations:** 1 Faculty of Medicine, Department of Psychiatry, Adiyaman University, Adiyaman, Turkey; 2 Can Sağlığı Foundation Contextual Behavioural Sciences Centre, Psychiatry, İstanbul, Turkey; 3 Faculty of Medicine, Department of Forensic Medicine, Adiyaman University, Adiyaman, Turkey

**Keywords:** Perpetrator, Childhood trauma, Intimate partner violence, Behavioral disorders, Psychological violence

## Abstract

**Purpose:**

Our study examines the socio-demographic, forensic psychiatric, and childhood trauma exposure (CTE) data of Turkish intimate partner violence (IPV) perpetrators and draws comparisons with the violence data.

**Methods:**

Data of male perpetrators referred to the domestic violence outpatient clinic by judicial authorities between November 2019 and June 2022 were retrospectively examined, with a focus on CTE data.

**Results:**

The mean age of the male perpetrators examined in the study was 37.1 years. Among the overall sample, 16.2% (*n* = 17) had experienced violence at school in childhood, and 22.9% (*n* = 24) had experienced CTE. Regarding the frequency of domestic violence in their households, of the perpetrators admitted to the clinic for IPV, 40% (*n* = 42) reported rarely, 43.8% (*n* = 46) sometimes, and 16.2% (*n* = 17) often engaged in violent acts. There is a significant relationship between the frequency of IPV and the level of CTE (χ2: 13.052, SD: 2, *p* = 0.001, Cramer’s V: 0.353). Similarly, individuals who witnessed domestic violence during childhood were found to commit partner violence more frequently (χ2: 8.157, SD: 2, *p* = 0.017, Cramer’s V: 0.279).

**Conclusions:**

In this study, we found a strong relationship between CTE and IPV. To the best of our knowledge, our study is only example that investigates the relationship between CTE and IPV in a Turkish sample.

## Introduction

In the last few years, intimate partner violence (IPV) and sexual violence against women have received increasing attention and recognition as global health issues. According to the report organized by the World Health Organization (WHO), one out of every three women in the world experiences is exposed to physical or sexual violence at least once in their lifetime (WHO 2021). This includes physical, psychological, and sexual violence, as well as insults, threats, and persistent pursuit by a current or former partner. Although IPV can be bidirectional, many aggressors are male, and victims are generally female (González-Álvarez et al. 2022).

Violence, especially against women, remains a pressing and ever-relevant issue, both worldwide and in Turkey, with its prevalence increasing day by day. The prevalence of IPV against women ranged from 67.7 to 85.4% in regional studies conducted in Turkey (Gencer et al. 2019; Gumus et al. 2020; Secgin et al. 2022). According to a 2009 report produced in collaboration with Hacettepe University and the Turkish Ministry of Family and Social Affairs, the Turkish state stopped publishing statistics on violence against women because the number of femicides increased thousands of times between 2002 and 2009. Since then, civil society organisations have shared records and statistics on violence against women (Turkish Republic 2009; Tekkas Kerman and Betrus 2020). Unfortunately, there is a lack of information on the prevalence of IPV across Turkey. In societies where violence against women is widespread, cultural elements and codes learnt and acquired in the family and environment play an essential role. Therefore, some cultural assumptions need to be questioned to evaluate these dynamics. In cultural and societal contexts, feminine traits are often ascribed to women, including qualities like compliance, submissiveness, and avoiding dissent. Conversely, men are socialized to exhibit assertiveness, take a prominent role, and assume leadership responsibilities (Saritas 2023). Another issue that needs to be addressed in IPV against women is patriarchal culture. In patriarchal societies like Turkey, men are seen as superior to women, and this cultural framework may encourage the perpetuation of violence (Dikmen and Munevver 2020).

The deleterious effects of violence extend beyond its immediate victims, impacting subsequent generations, as well. Violence affects all family members, with direct and indirect effects, particularly on children. Exposure to violence within the family during childhood, either directly or as a witness, has far-reaching implications, as it can impact interactions in other socialization contexts. Recent meta-analyses have shown a positive correlation between CTE and IPV perpetration (St-Pierre Bouchard et al. 2023; Li et al. 2020; Kotan et al. 2020). Violence, therefore, is not only an issue for individuals or groups but also emerges as a social issue. Learning violence through imitation in childhood has created in men the idea of gaining power over others through violence. At the same time, inadequate conflict and problem-solving skills, anger control problems, failures in education, over-sensitivity and jealousy have led to the development of violence tendency (Cengisiz and Nehir 2023; Caliskan and Cevik 2018). Furthermore, children who experience interpersonal trauma during childhood are more likely to experience anger, depression, and anxiety (Turner et al. 2006). Negative, inconsistent and oppressive parental behavior has also been reported to contribute to IPV exposure and perpetration whereas high parental support was found to reduce IPV (Mcleod et al. 2020; Miller et al. 2009).

Studies examining IPV perpetrators have found that men with antisocial, impulsive personality traits tend to engage in high levels of IPV, while men with no or minimal psychopathology and no formidable background are less likely to resort to violence. (Holtzworth-Munroe 2005; Capaldi et al. 2012) Additionally, research has shown that a range of mental disorders, including depression, anxiety disorders, substance use disorder, and personality disorders, are significant risk factors for men’s perpetration of violence against women (Spencer et al. 2019; González et al. 2016; Yu et al. 2019). In addition, various individual factors (personality structure and susceptibility to mental disorders) and environmental confounding factors (parental attitudes, socioeconomic factors, and social relationships) were found to contribute to IPV (Capaldi et al. 2012; St-Pierre Bouchard et al. 2023). In line with these, a study conducted on randomly selected married men in Turkey reported that 30.5%,30.5%, 17.1%, 6.7%, and 5.7% of the reasons for committing IPV were economic problems, relationship issues, psychological problems, substance abuse, and societal pressure, respectively (Genc et al. 2019).

To the best of our knowledge, despite the existence of longitudinal studies in the literature, there is no study in Turkey that investigates the childhood traumatic experiences of IPV perpetrators along with other IPV perpetration risk factors. We believe that presenting a sample from Turkey, where violence against women is prevalent, will offer a valuable culturally sensitive contribution to the existing literature. The objectives of our study are as follows:

1. To examine the sociodemographic and forensic psychiatric data of IPV perpetrators.
2. To investigate the relationship between childhood traumatic experiences, and other IPV perpetration risk factors.

Building upon the literature discussed above (St-Pierre Bouchard et al. 2023; Li et al. 2020; Kotan et al. 2020; Cengisiz and Nehir 2023; Caliskan and Cevik 2018), discussed above, our hypothesis posits a correlation between exposure to or witnessing childhood trauma and intimate partner violence in a Turkish sample.

## Materials and methods

### Study design

In accordance with the decision dated 03/06/2022 and numbered 2022/6–6, this retrospective descriptive study was conducted with local ethics committee approval in compliance with the Helsinki Declaration of 1975, as revised in 2008. The study was conducted from data of individuals who were referred to a university hospital’s “Domestic Violence Outpatient Clinic” between November 2019 and June 2022. The Domestic Violence Outpatient Clinic was established within the university hospital’s psychiatry clinic as part of the “Dealing with Domestic Violence” protocol signed in 2019 by local authorities (Public Prosecutor’s Office, University Hospital, Directorate of Family and Social Services). In addition, the domestic violence outpatient clinic is the first outpatient clinic aims to contribute a pilot project in Turkey. In this context, 196 domestic violence cases, whose judicial investigations were ongoing by local judicial authorities, were referred to the center for psychiatric evaluation between November 2019 and June 2022.

### Data collection procedure

Individuals referred to the Domestic Violence Outpatient Clinic are evaluated by a psychiatrist regarding the risk of domestic violence. The factors that may increase the risk of domestic violence are routinely evaluated, including the individual’s criminal and medical records, psycho-active substance use, statements related to the violent incident, and the way the incident occurred. Simultaneously, psychiatric evaluation is conducted, considering factors such as psychotic disorders, mood disorders, personality disorders, the presence or history of psychoactive substance use, childhood traumas, and the quality of relationship, among other parameters. After assessing these factors, a psychiatric risk for the recurrence of violence is provided. To access these factors for risk assessment, the Non-Interventional Ethics Committee of [University name was blinded for peer review] University decided that informed consent was not required from the perpetrators, as the data for the study were collected retrospectively.

The current study involves analyzing records related to these reports, specifically examining whether individuals experienced or witnessed any trauma, neglect, or abuse and whether they had behavioral problems during childhood. We also noted relationship characteristics such as frequency of intimate partner violence and marital conflicts, relationship satisfaction, and medical and forensic data for analysis.

Beyond above variables, the psychiatric conditions of individuals at the time of psychiatric examination were recorded based on the International Classification of Diseases, 11th Revision (ICD-11) criteria because in the Turkish health system, diagnosis codes are organized according to ICD-11.

### Participants

Data of people who were referred to a university hospital’s “Domestic Violence Outpatient Clinic” between November 2019 and June 2022 were examined retrospectively. During the Covid-19 pandemic the outpatient clinic was closed for almost six months due to restrictions.

**Fig. 1:**
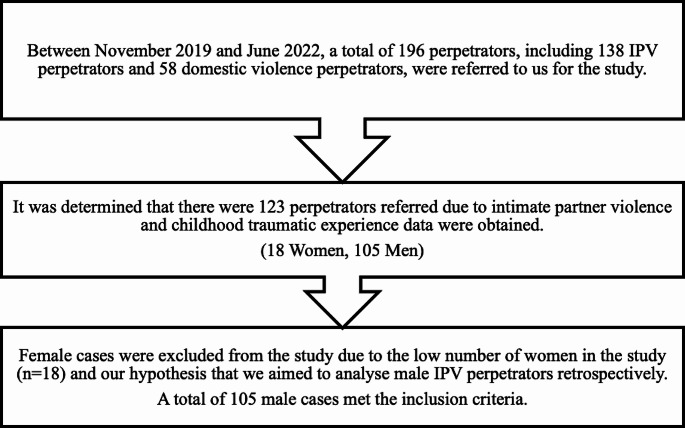
Flow-chart of the study enrolment protocol

Between November 2019 and June 2022, 196 perpetrators were referred by the judicial authorities to the domestic violence outpatient clinic. Of these, 58 were excluded from the study due to the absence of intimate partner violence. The childhood traumatic experiences of 15 out of 138 IPV perpetrators could not be obtained. Childhood traumatic experience data of 123 perpetrators who were referred to the domestic violence outpatient clinic due to IPV were obtained from records. In accordance with our hypothesis, we excluded 18 female cases from the study due to their small number. The flow chart of the study enrollment protocol is provided in Fig. 1.

The inclusion criteria for the study sample are as follows: availability of data on childhood traumatic experiences and forensic reports, absence of severe psychiatric disorders (such as schizophrenia or mental retardation) or neurological diseases (such as dementia or cerebrovascular disease), being over 18 years of age, male and a perpetrator of IPV. Cases were excluded from the study if we lacked accessible data on their childhood traumatic experiences and forensic reports, had severe psychiatric disorders (such as schizophrenia or mental retardation) or neurological diseases (such as dementia or cerebrovascular disease), were under 18 years of age, female, or not perpetrators of IPV.

### Data analysis

The categorical variables in the study were given as frequency and percentage while the descriptive statistics and continuous variables were given as mean standard deviation. Categorical variables were grouped, and their percentages were calculated. Pearson’s chi-square was used to compare frequencies according to compatibility. Cramer V was reported in effect size measure for the chi-square test. One-way ANOVA test was used for comparison of numerical data between three or above independent groups. All statistical analyzes, tables, and figures were prepared using SPSS 22 (IBM Corp, Armonk, NY) program. The variables with a p value of < 0.05 were considered significant and the existence of a relationship between the variables was statistically demonstrated.

## Results

A total of 105 male perpetrators were included in this study. The mean age of the cases was 37.10 ± 7.75. The majority of the perpetrators (52.4%) were aged 31–40 years. In our study, 90.5% (*n* = 95) of all cases were married, and 39% (*n* = 41) had a high school education or higher. Only 3.8% (*n* = 4) of perpetrators were unemployed. However, 49.5% (*n* = 52) of perpetrators reported a low economic situation. Frequencies of other sociodemographic and forensic psychiatric data of perpetrators of intimate partner violence are shown in Table 1.

**Table 1:** Frequency values of sociodemographic and forensic psychiatric data of perpetrators of intimate partner violence

Variable		*n*	%
**Age**	18–30	20	19.0
	31–40	55	52.4
	41–50	24	22.9
	51–60	6	5.7
**Occupation**	Unemployed	4	3.8
	Laborer	31	29.5
	Civil servant	22	21.0
	Self-employed	13	12.4
**Education**	Illiterate	2	1.9
	Literate	3	2.9
	Primary school graduate	33	31.4
	Middle school graduate	26	24.8
	High school graduate	25	23.8
	University graduate	16	15.2
**Marital status**	Married	95	90.5
	Single	5	4.8
	Divorced	5	4.8
**Economic status**	Low	52	49.5
	Medium	33	31.4
	High	20	19.0
**Self-mutilation**	Yes	19	18.0
	No	86	82.0
**Suicide attempt**	Yes	15	14.3
	No	90	85.7
**Problems with anger control**	Yes	14	13.3
	No	91	86.7
**Type of Intimate Partner Violence**	Physical violence	49	46.7
	Psychological violence	25	23.8
	Physical and Psychological violence	31	29.5

In our study, 13.3% (*n* = 14) of perpetrators were found to have anger control problems and the majority of cases (46.7%) were referred with a suspicion of physical violence (Table 1). Perpetrators with anger control problems and perpetrators who perpetrated physical and psychological violence together, had a significantly higher frequency of partner violence. (χ2: 8.582, SD: 2, *p* = 0.014, Cramer’s V: 0.286; χ2: 9.796, SD: 4, *p* = 0.044, Cramer’s V: 0.216 respectively).

When analyzing the data on CTE of the perpetrators in our study, it was found that 22.9% (*n* = 24) of the perpetrators were exposed to domestic violence in childhood, and 26.6% (*n* = 28) witnessed domestic violence in childhood. Among the cases, 16.2% (*n* = 17) had experienced violence at school in childhood, and 37.1% (*n* = 39) had low academic performance in childhood. It was found that 33.3% (*n* = 35) of perpetrators experienced sensitivity, irritability and anger in childhood, 30.5% (*n* = 32) were frequently involved in fights as children and 13.3% (*n* = 14) possessed sharp objects in childhood. In addition, it was found that 35.2% (*n* = 37) of the cases had a strict family attitude in childhood, and 16.1% (*n* = 17) had an introverted personality structure in childhood. Furthermore, 37.1% (*n* = 39) of the perpetrators in our study had low academic performance in childhood. At the same time, 33.3% (*n* = 35) had a previous criminal record, and 11.4% (*n* = 12) were found to have used substances in childhood (Table 2).

**Table 2:** Comparison of childhood trauma exposure data with IPV frequency data

Variables		Frequency of IPV				
		Rarely	Sometimes	Often	p	Effect size^*^
**Exposure to domestic violence during childhood**	Yes	3 (12.5%)	18 (75.0%)	3 (12.5%)	**0.001**	**0.353**
	No	39 (48.1%)	28 (34.6%)	14 (17.3%)		
**Witnessing domestic violence during childhood**	Yes	7 (25.0%)	12 (42.9%)	9 (32.1%)	**0.017**	**0.279**
	No	35 (45.5%)	34 (44.2%)	8 (10.3%)		
**Experiencing violence at school in childhood**	Yes	3 (17.6%)	10 (58.8%)	4 (23.6%)	0.119	0.201
	No	39 (44.3%)	36 (40.9%)	13 (14.8%)		
**Childhood sensitivity**,** irritability**,** and anger**	Yes	11 (31.4%)	16 (45.7%)	8 (22.9%)	0.294	0.153
	No	31 (44.3%)	30 (42.9%)	9 (12.8%)		
**Fighting often as a child**	Yes	11(34.4%)	11 (34.4%)	10 (31.2%)	**0.021**	**0.272**
	No	31 (42.5%)	35 (47.9%)	7 (9.6%)		
**Have a strict family attitude in childhood**	Yes	7 (18.9%)	19 (51.4%)	11 (29.7%)	**0.001**	**0.359**
	No	35 (51.5%)	27 (39.7%)	6 (8.8%)		
**The condition of being introverted as a child**	Yes	3 (17.6%)	9 (52.9%)	5 (29.5%)	0.078	0.221
	No	39 (44.3%)	37 (42.0%)	12 (13.7%)		
**Possession of sharp objects in childhood**	Yes	6 (42.9%)	5 (35.7%)	3 (21.4%)	0.760	0.072
	No	36 (39.6%)	41 (45.1%)	14 (15.3%)		
**Academic performance in childhood**	Low	7 (17.9%)	23 (59.0%)	9 (23.1%)	**0.002**	**0.347**
	High	35 (53.0%)	23 (34.8%)	8 (12.1%)		
**Substance use in childhood**	Yes	4 (33.3%)	5 (41.7%)	3 (25.0%)	0.666	0.088
	No	38 (40.9%)	41 (44.1%)	14 (15.1%)		
**Criminal history**	Yes	12 (34.3%)	16 (45.7%)	7 (20.0%)	0.624	0.095
	No	30 (42.9%)	30 (42.9%)	10 (14.3%)		

^*^ Cramer V value was reported in effect size measure

Regarding the frequency of domestic violence in their households, of the perpetrators admitted to the clinic for IPV, 40% (*n* = 42) reported rarely, 43.8% (*n* = 46) sometimes, and 16.2% (*n* = 17) often engaged in violent acts (Table 2). An examination of the relationship between age and violence frequency did not reveal any significance (*p* = 0.966). Similarly, no significant relationship was identified between economic status and frequency of IPV (*p* = 0.966). In our study, when perpetrators’ childhood trauma data were compared with IPV frequency data, there was a positive and significant association between experiencing and witnessing violence in the family during childhood and IPV frequency, as shown in Table 2. Additionally, a significant association was observed between frequent fighting in childhood, having a strict family attitude, and low academic performance in childhood with IPV frequency. The comparison of other CTE data with IPV frequency is presented in Table 2. It was also found that perpetrators with low academic performance in childhood were significantly more likely to have been exposed to and witnessed domestic violence in childhood (χ2: 8.568, SD: 1, *p* = 0.003, Cramer’s V: 0.286; χ2: 12.049, SD: 1, *p* = 0.001, Cramer’s V: 0.339 respectively). Moreover, those who had experienced or witnessed domestic violence within the family during childhood were found to experience significantly higher sensitivity, irritability, and anger (χ2: 6.076, SD: 1, *p* = 0.014, Cramer’s V: 0.241; χ2: 7.159 SD: 1, *p* = 0.007, Cramer’s V: 0.261).

When comparing childhood trauma exposure data and types of violence, it was found that those who were exposed to domestic violence in childhood were more likely to use both physical and psychological violence than those who were not (χ2: 5.134, SD: 2, *p* = 0.047, Cramer’s V: 0.221). In addition, those who engaged in physical and psychological violence toward their partners were found to have lower academic performance (χ2: 13.707, SD: 2, *p* = 0.001, Cramer’s V: 0.361). Similarly, a significant relationship was found between exposure to violence during school years and the type of violence (χ2: 5.566, SD: 2, *p* = 0.062, Cramer’s V: 0.230). Perpetrators with introverted tendencies were found to engage in more frequent physical and psychological violence compared to those without such tendencies (χ2: 6.095, SD: 2, *p* = 0.047, Cramer’s V: 0.241). Perpetrators who had access to sharp objects during childhood were more likely to engage in physical and psychological violence toward their partners (χ2: 9.630, SD: 2, *p* = 0.008, Cramer’s V: 0.303).

Among the intimate partner perpetrators, 33.3% (*n* = 35) had a history of mental disorders. Among those with mental disorders, 12.4% (*n* = 13) had substance use disorders, 5.8% (*n* = 6) had personality disorders, 3.8% (*n* = 4) had anxiety disorders, and 2.9% (*n* = 3) had depression. The presence of mental disorders was associated with an increased frequency of partner violence (χ2: 6.259, SD: 2, *p* = 0.044, Cramer’s V: 0.244). The presence of mental disorders was compared with childhood experiences of abuse, witnessing abuse and frequent childhood arguments, but no significant associations were found.

## Discussion

In this study, we retrospectively assessed childhood traumas among IPV perpetrators mandated to undergo psychiatric evaluation by legal authorities following IPV incidents. was found that individuals who had experienced violence and abuse within their families and during their school education in childhood were more likely to engage in IPV during adulthood. In adulthood, there is a positive association between intimate partner violence (IPV) and several factors observed earlier in life, including low academic achievement during childhood and adolescence, exposure to violence, frequent irritability and anger, growing up in a repressive family environment, and exhibiting an introverted personality pattern. Although it is known that psychological violence is more common among women who exposed to IPV (Capaldi et al. 2012; Ahnlund et al. 2020), we found that physical violence was more common in our study. We believe this is because the cases we studied were referred by the judicial authorities as having perpetrated domestic violence. It may also be the result of women perceiving physical abuse as more serious than psychological abuse (Wilson and Smirles 2022). The most striking aspect of our study is that contrary to the existing literature (Capaldi et al. 2012; González-Álvarez et al. 2022; Singh et al. 2014), mental disorders were less common among perpetrators (33.3%).

When examining the relationship between age and IPV, several studies in the literature have suggested that older age is a protective factor against IPV (Capaldi et al. 2012; Kim et al. 2008; Rodriguez et al. 2001). In a study conducted with IPV perpetrators in Spain (González-Álvarez et al. 2022), the average age was 39.7, while another study reported an average age of 37.3 (St-Pierre Bouchard et al. 2023). In our study, the mean age of perpetrators was approximately similar to the literature at 37.10. Although the impact of education on IPV perpetration varies across geographical areas and population groups, higher levels of education are generally associated with both perpetration and victimization (Cunradi et al. 2002; Mannell et al. 2022; Alkan and Tekmanlı 2021). In a prospective study, low verbal intelligence was found to be the only developmental factor predicting IPV in men (Lussier et al. 2009). Although some studies (Kliem et al. 2019; DePrince et al. 2009) suggest that low academic achievement increases the risk of witnessing IPV and future IPV, as in our study, other studies (Stiller et al. 2022; Capaldi et al. 2012) argue the opposite. In addition, in our study we found that perpetrators with poor school performance were more likely to perpetrate violence against their partners, and those who perpetrated psychological and physical violence together had lower school performance than those who perpetrated only one type of violence. We believe it is difficult to explain the relationship between IPV and education because of confounding factors. There are studies that link a low level of education as well as a low economic level to IPV (Vyas and Watts 2009; Caetano et al. 2008). In this study, however, no significant relationship was found between economic status, occupation, and violence. In Turkish society, individual factors, the environment in which one lives, and entrenched patriarchal patterns play a decisive role in women’s social status. We suspect that several abused women do not report their experiences to legal authorities, and, perhaps, the lack of statistical significance is due to women with financial independence being more likely to file complaints.

Studies in the literature have linked traumatic childhood experiences with perpetration of IPV (Chowdhury & Heuveline 2023; McKinley et al. 2021; Voith et al. 2020). A study of university students in Turkey found that those with a history of childhood domestic violence were more likely to perpetrate partner violence (Gokkaya and Ozturk 2021). In another study, adolescents’ exposure to peer bullying at school, in addition to domestic violence, were reported as a factor that facilitates IPV (Vural et al. 2020). In our study, similar to the existing literature, offenders who experienced family violence were found to be more likely to perpetrate IPV, and those who experienced violence during their school years were found to be more likely to perpetrate both psychological and physical forms of violence. Various hypotheses have been proposed regarding why individuals who have experienced childhood traumas engage in IPV during adulthood (St-Pierre Bouchard et al. 2023). Deficits in social problem-solving skills (Mumford et al. 2019) and hostile attitudes towards the environment (Zhu et al. 2020) have been observed in perpetrators exposed to parental rejection and abuse during childhood.

White and Widom pointed out that hostility, problematic behavior, and harsh parental attitudes were consistent risk factors for adult engagement in IPV (White and Widom 2003; Skandro et al. 2023). In our study, similar to the literature, it was found that perpetrators who had experienced childhood trauma were more hypersensitive, angry and irritable. In addition, IPV has been found to be more prevalent in individuals who grow up in oppressive and authoritarian family environments (Sezer et al. 2020). The oppressive and authoritarian family attitude causes the individual to perceive life as a constant threat, to lose the ability to distinguish safe environments, and to prevent the development of regulatory processes (Sezer et al. 2020; Voith et al. 2020). In Turkish society, the prevalence of an authoritarian family structure often leads to the implantation of strict rules in a child’s mind, thus normalizing violent and aggressive behavior in adulthood. In this context, violence often takes on the role of mediator in unresolved conflicts (Ulker 2023). Similarly, in our study it was found that perpetrators with strict family attitudes during childhood were more likely to use violence. We believe that attitudes that legitimize IPV are higher among individuals who have grown up in family environments where the oppressive and authoritarian structure of the patriarchal system is felt.

According to Katz, perpetrators/fathers are generally less interested in their children and less supportive of free expression and creativity in their children’s lives (Katz 2015). Decreased participation of children in social activities may contribute to emotional and behavioral problems in the future. The perpetrator father can be a bad role model in relationships and conflict resolution. The presence of a violent father can negatively affect the relationship between mother and child, prevent children from spending quality time with their mothers, and contribute to children’s emotional and behavioral problems (Katz 2016). The witnessing of threats and violence between parents is considered a contributing factor to both becoming a victim and perpetrator of violence (Caetano et al. 2008). In Turkey, the rate of witnessing domestic violence among young people aged 12–21 years was found to be 17%. It was observed that boys who witnessed violence repeatedly tended to use violence in the face of stress, identified with the violent father and perceived violence as a natural situation (Almis et al. 2020). Similarly, in our study, witnessing violence between parents also led to an increase in the frequency of IPV. The concept of substituting a victim with a perpetrator and the fluidity of roles within partner relationships are perceived as interconnected, and this behavioral pattern often repeats itself in adulthood (Forke et al. 2018; Gilbar et al. 2020).It would be a positive step to identify and make some interventions in childhood to prevent these children who witness violence, whose self-confidence has decreased and whose ego has been eroded from having problems in their relationships in their later lives. The findings of this study also show that children can benefit from this.

Mental disorders, especially substance use and personality disorders, are associated with an increased risk of IPV perpetration (Yu et al. 2019). Antisocial behaviors at ages 17–18 facilitate the perpetration of IPV in adulthood (Capaldi et al. 2001; Huesmann et al. 2009). In our study, although the diagnosis of antisocial personality disorder was not precisely established, the use of sharp and piercing instruments and frequent childhood fights were found to be associated with IPV. Another condition that predisposes to crime is having an introverted personality trait that enjoys solitude. In a study conducted in Turkey, loneliness was found to play a partial mediating role in the relationship between CTE and attitudes towards crime (Pomakoglu 2023). In our study, we found that introverts were more likely to perpetrate both physical and psychological violence together during childhood. In our study, the relatively low incidence of mental disorders among perpetrators can be attributed to the inclusion of only those cases that sought assistance due to legal mandate. Furthermore, we believe that society’s perspective on violence also plays a role in the prevalence of this problem. According to a study investigating violence in societies dominated by Arab and Islamic cultures, although violence is not inherent in the Islamic ideology, it is attributed to culture and traditions (Douki et al. 2003). It may be related to women’s perceptions of violence, briefly, women may not be aware that the behaviors they witness constitute violence.

### Clinical implications

One of the strengths of this study is that it is the first study in Turkey to examine the sociodemographic, childhood traumatic experiences and behavioral problems data of male IPV perpetrators. In Turkey, perpetrators of violence against women were found to be associated with IPV if they had experienced or witnessed violence during their childhood, education, or family life. In addition, we found that Turkish male perpetrators who experienced authoritarian, strict family attitudes during childhood were strongly associated with violence. In Turkey, the position of men within the family structure and the responsibilities placed on them from childhood are of great cultural importance. In Turkish families, men are expected to be dominant in the home and the husband is seen as the official authority of the family (Saritas 2023; Dikmen and Munevver 2020). The cultural codes resulting from the authoritarian and oppressive approach of the patriarchal system pave the way for male children to become perpetrators of violence in adulthood. Our findings support the need to challenge some of the cultural assumptions prevalent in Turkish society and to provide cognitive, ideological and behavioral interventions for perpetrators. Furthermore, our clinical data indicate that the relationship between psychiatric disorders and IPV perpetration in Turkish society is quite different from the existing literature (Capaldi et al. 2012; González-Álvarez et al. 2022; Singh et al. 2014), In this context, our findings point to the existence of beliefs and attitudes that legitimize violence in Turkish society.

### Future research directions

Although there are studies on violence against women in Turkey (Arabaci and Uygun 2022; Cengisiz and Nehir 2023), these studies either focus on the general population or do not include data on childhood traumatic experiences. Our study has extensively examined data on IPV and childhood traumatic experiences. Future studies should focus on qualitative research with male perpetrators of IPV to better understand violence against women in Turkey and to develop effective intervention programmes. There is a need for mixed methods studies, particularly focusing on childhood traumatic experiences, to better understand their impact on IPV perpetration. Moreover, research endeavors should undertake comprehensive qualitative investigations into the impact of Turkish culture on male perpetrators of violence against women. Longitudinal studies with more data can be conducted after the widespread establishment of domestic violence clinics for perpetrators. In addition, the effectiveness of domestic violence clinics in reducing violence should be demonstrated, along with data on the outcomes of interventions.

### Limitations

Our study had some limitations. This retrospective study subjectively assessed childhood trauma questions without the use of standardized scales. The lack of childhood trauma scales in our study makes it difficult to standardize the data and may affect the generalizability or comparability of the findings. Furthermore, the lack of qualitative techniques in our study hinders a more flexible and in-depth examination of our findings. One of the limitations of our study is that the number of participants with a history of childhood traumatic experiences was low. Female perpetrators were excluded from the study due to the majority of participants being male. The limited data in our study is due to the recent establishment of domestic violence clinics and intervention programmes in collaboration with judicial authorities. The lack of study data may limit the generalizability of our findings to Turkish society. We believe that several cases of violence perpetrated against women in Turkish society go unreported because women fear stigmatization. In the context of our study, relatively low frequency of psychiatric diagnoses and CTE could potentially be linked to perpetrators’ inclination to provide biased responses during questioning. This bias may arise from the secondary benefits associated with complying with legal authorities’ mandates to seek assistance.

## Conclusions

Our findings confirm a strong relationship between CTE and IPV. To the best of our knowledge, our study is the first project for IPV perpetrators carried out in cooperation with judicial authorities in Turkey. Due to the lack of previous IPV perpetrator behavior change programs in Turkey, it is thought that there is a need for special services for perpetrators of violence. We believe that it is essential to inquire about whether IPV perpetrators who are legally mandated to seek assistance have experienced childhood trauma and implement cognitive-behavioral intervention programs to help them improve their communication capabilities and gain conflict resolution skills with a focus on their traumas. Furthermore, we believe that when men, who are prone to violence, seek assistance, our findings can facilitate the identification of situations with a high risk of violence.

## Data Availability

The data created within the scope of the study can be accessed from the authors upon appropriate request, provided that legal and ethical rules are respected.
